# Using a simulation-based approach to promote structured and interactive nursing clinical handover: a pre- and post-evaluation pilot study in bilingual Hong Kong

**DOI:** 10.1186/s12912-023-01189-w

**Published:** 2023-02-10

**Authors:** Jack Pun

**Affiliations:** Department of English, City University of Hong Kong, 83 Tat Hong Avenue, Kowloon Tong, Hong Kong

**Keywords:** Handover, Simulated-based training, Communication, Nursing, Patient safety, Bilingual, Hong Kong

## Abstract

Effective clinical nursing handover involves the transfer of responsibility and accountability for patient care between nurses, leading to better patient safety and continuity of care. Nurses in bilingual contexts, such as Hong Kong – where nurses are trained in English but communicate in Cantonese – may find it challenging to deliver a safe clinical handover. This article reports a pilot study in which a simulation-based approach is being developed to enhance nursing handover with structured and interactive interactions, using handover protocols such as ISBAR (introduction, situation, background, assessment, recommendation and readback) and CARE-team (connect, ask, respond, empathise) protocols in a bilingual context. The study has a pre‐ and post-evaluation design involving a questionnaire survey before and after a 4‐hour workshop. Fourteen selected bilingual nurses in Hong Kong were trained according to the ISBAR and CARE-team protocols, and their perceptions of complete and structured handovers were evaluated before and after training using the questionnaire. The nurses reported that they were more self-confident in their handover experiences, with a deeper understanding of ISBAR and CARE-team protocols after the simulation-based training intervention, leading to better (i.e., more structured and interactive) clinical handover between nurses. Overall, the staff perceived their handover communication to have improved using simulation-based training.

**Supplementary Information:**

The online version contains supplementary material available at 10.1186/s12912-023-01189-w.

## Introduction

Clinical handover between nurses is fundamental to the effective transfer of patient care plans between healthcare providers. Handover communication represents a transition point of responsibility and accountability for patient care [1–3]. An effective clinical handover is characterised by elements of good practices leading to better patient care and safety [4–6]. First, nursing staff should be reminded about the transfer of responsibility and accountability for patient care. The use of a standardised structure (i.e., handover protocols) can facilitate the accurate and effective communication of crucial information between staff [7, 8]. Second, the handover should have a clear structure. A designated individual should take a leading role with responsibility for ensuring the exchange of all relevant communication in a timely manner. Third, a high level of involvement of the patient and caregiver allows clinicians to obtain information that is not necessarily otherwise available. Nurses can listen and obtain an understanding of the information provided, thereby ensuring the healthcare progress, treatment options and care plan of the patient. Fifth, thorough preparation is needed before the handover is carried out. Nurse managers should assign their staff to carry out handovers at an allocated time and venue that all essential staff can attend. A verbal handover supported by documentation such as a handover sheet is recommended [9]. The above elements allow nurses to provide an accurate and timely transfer of patient care. Clinical handover has been highlighted as a point of vulnerability in the provision of a patient’s care. Problems concerning the transfer of patient information can result in workflow inefficiencies or even medical incidents [10]. In a bilingual context, where nursing staff need to use the two languages (i.e. first language and English) in both verbal and written forms to exchange information for clinical purposes, this may create a complicated process of ‘translanguaging’ at clinical handovers, which could be time-intensive, difficult to monitor and susceptible to errors (See more [11–15]). To address this gap, we need a suitable protocol to standardise staff’s translanguaging processes to ensure a safe and efficient handover process in a bilingual environment. Therefore, the aim of this study’s simulation-based approach to communication training is to build awareness of good handover practices by using the ISBAR (introduction, situation, background, assessment, recommendation and readback) [16] and CARE-team (connect, ask, respond, empathise) protocols [5] to facilitate effective clinical handovers in nursing.

These two protocols were situated under the construct of a simulation-based training technique, which is a widely-adopted approach to improve healthcare quality by building up simulated scenarios that is “representative of actual operational conditions” (p. 364) for medical and nursing personnel to practise, develop, and refine requisite skills in real clinical settings [17]. Research has been long providing statistical evidence of its more significant positive effects than other approaches in terms of improving participants’ clinical knowledge, skills, and self-efficacy in various scenarios, including handover [18–20]. Moreover, comparing with other approaches, such as group discussing, debriefing, peer learning, and lecturing, the simulated-based approach is especially different in creating a realistic, interactive, and communicative healthcare setting for trainees to repeatedly engage in and practice skills without worrying about causing harm to patients and making mistakes [16]. In this sense, this approach can be suitable for the implementation of ISBAR and CARE-team protocols to practice trainees’ communication abilities with colleagues and patients during clinical handover [18, 21].

## Method

### Research design

This study adopted a pre‐ and post-evaluation design to conduct simulation-based handover training for bilingual nurses in Hong Kong. The participants’ perceptions of clinical handover practice were evaluated before and after the training.

### Participants

Nursing staff at two government-funded hospitals in Hong Kong were recruited to participate in this study using convenience sampling technique. We recruited fourteen nurses working in a bilingual environment in which they were trained in a second language (English) but communicate in their first language (Cantonese) in their routine daily work. The participants had not previously received any communication training or professional development workshops on clinical handover.

### Ethics

Ethical approval was obtained from the human ethics sub-committee of the first author’s institution prior to conducting the study. Nursing staff who expressed interest in participating were formally informed about the purpose of the study and their rights. Written consent was obtained for participation.

### Procedures

The simulation-based handover training consisted of four stages: needs analysis, training development, implementation and evaluation. The ISBAR and CARE-team protocols were integral to the training. They were explained in greater detail below (refer to Protocols section).

### Needs analysis

In 2019–21, the research team observed, recorded and analysed nursing handovers at a hospital in Hong Kong to assess current practice and to recommend areas of development for a simulation-based nursing handover training programme aimed at addressing the communication challenges encountered by nursing staff in a bilingual context [7, 11]. The team comprised a communication researcher, clinicians, hospital management, senior nursing staff and nursing professors. We spent two days observing and audio-recording shift-change nursing handovers in one medical and one surgical ward. These recordings were then transcribed, translated into English and analysed to identify key content areas for developing simulation-based handover training. The team also consulted with stakeholders such as ward managers, nurse educators and senior nurses about their views on the observed handover experiences and collected examples of both formal and informal handover documentation. All of this data collection took place between 1 November and 31 December 2021.

The collected materials and observations informed the development of a structured and interactive nursing clinical handover training. In collaboration with hospital management, the team identified the simulation approach that would be the most suitable delivery method for promoting effective handover practice to the nursing staff. The team also identified two main areas for training in which changes to handover practice would likely enhance patient safety and continuity of care: using an ISBAR protocol to ensure that nurses conduct handovers in a sequential and logical manner, resulting in better patient safety, and using a CARE-team protocol to promote better quality interactions, leading to better comprehension and continuity of care.

### Development of training

The training was developed according to the above needs analysis of the bilingual clinical context, extensive local and overseas studies and the principles of deliberate practice (see [12]). The training modules and materials incorporate re-enacted videos of anonymised interactions, role-play simulations and a framework of communication protocols, checklists and practical communication strategies. The programme is evidence-based, with authentic data from nursing staff, and translates communication theories to influence the actual practice of nursing professionals. The intervention consisted of communication training that emphasised patient-centeredness and patient safety for better clinical handover, with more interaction and logical sequences. A 4-h communication training programme focused on better logical sequencing when transferring patients’ information (ISBAR) and better quality of interaction for patient safety (CARE-team) at clinical handover for nursing staff was developed and evaluated. The shift-to-shift handover practices of the participants after this training were assessed using a validated questionnaire and their recorded handover performance during the workshop.

### Protocols

The two recognised handover protocols used in this study (ISBAR and CARE-team) can help nurses, especially those working in a bilingual context (see [13]), with both the informational and interactive aspects of the process (see [8]). The informational dimension relates to the content of messages, i.e., what nurses should say relating to the structure and logical sequences of a handover. The ISBAR handover protocol helps nurses to improve the informational dimension of a handover, ensuring that the message is well structured with a logical flow (i.e., what to say and ask). As described in Eggins et al. [8], ISBAR stands for (a) **identification**: the patient and nursing staff responsible for the handover; (b) **situation**: the patient’s presenting condition; (c) **background**: the patient’s medical history and social background; (d) **assessment**: the patient’s current condition and intraoperative issues; (e) **actions**: the actions performed for the patient; (f) **recommendation**: the treatment plan and instructions for the next shift; (g) **readback**: accountability and responsibility for ongoing care tasks. It suggests an incoming nurse receiving handover, repeating back the information they received, and confirming they have interpreted the handover information correctly. Table 1 summarises the aims of each stage of the protocol.

**Table 1:** Summary of the ISBAR protocol for nurses (adapted from Eggins et al. [8])

Stage		Aim(s)	Time
**I**	Identification	Introduce yourself by stating the role you have played in this patient’s care	**Past**
		Clearly and accurately identify and locate the patient	
		Identify the doctors responsible for the patient’s care	
**S**	Situation	Explain the patient’s presenting condition	
**B**	Background	Hand over the patient’s medical and social background relevant to this admission	
**A**	Assessment	Succinctly describe the patient’s general condition, clinically and behaviourally, during your shift	**Present** (your shift, outgoing nurse)
		Provide key clinical observations/data	
	Actions	State what you have done for the patient on your shift	
**R**	Recommendations	Explain the treatment plan for this patient	**Future** (your colleagues, incoming nurse)
		Explain the care plan and actions required of nursing staff	
	Readback	Clearly hand over accountability and responsibility for ongoing care tasks	

The ISBAR protocol incorporates transition markers that can help bilingual nurses to ensure the accuracy and quality of information presented through the use of signposting when starting or finishing each ISBAR stage or substage. Using transition markers, colleagues can know what stage of the handover has been reached. A list of examples of transition markers at each stage of the protocol can be found in the  Additional file 1: Appendix.

The CARE-team protocol is interaction-based and prioritises nurses’ engagement and patients’ participation, which in turn promote the quality and consistency of nursing handovers [7]. In the handover, an incoming nurse thus should participate actively by asking questions, confirming information and accepting responsibility. Incoming nurses can check that outgoing nurses have provided all of the correct and relevant information. Outgoing nurses can make sure that the incoming nurses know exactly what to do to care for the patient during the next shift. According to Eggins et al. [8], the CARE-team protocol consists of four steps that nurses should take: (1) **connect**: introduce themselves and clearly explain the purpose of the handover; (2) **ask**: gather medical details; (3) **respond**: react appropriately to others’ enquiries and contributions; and (4) **empathise**: recognise colleagues’ professional capabilities and individual needs, and respect patients’ needs for privacy, comfort and involvement. Table 2 presents a summary of the protocol with examples of each stage.

**Table 2:** CARE-team protocol (adapted from Eggins et al. [8])

Stage	Aim(s)
**Connect**	Outgoing nurse should greet the team, check who will be caring for the patients and make sure that all team members have a worksheet
**Ask**	Incoming nurses should be attentive and alert
	Outgoing nurse should ask incoming nurses if they have any questions or would like to go over any of the information at the end of the handover of each patient
	Incoming nurses should ask questions at the end of each ISBAR stage during the handover
**Respond**	Outgoing nurse should provide a succinct summary at the end of each patient’s handover
	Incoming nurses should restate the summary in their own words
**Empathise**	Outgoing nurse should respond to questions and react positively to whatever colleagues ask
	Incoming nurses should be attentive and actively ask questions and confirm comprehension

### Implementation and evaluation

The Nurses Handover Perceptions Questionnaire (NHPQ) was used to evaluate the participants’ perceptions and practices surrounding handovers before and after a 4-h training workshop at the participating hospital. The participants recruited were from two large regional public hospitals were invited to join the programme. We invited the participants to complete the NHPQ before and after the training workshop. We adapted the NHPQ survey from a previous study (Pun et al., 2020) that explored nurses’ perceptions of their handover experiences in Hong Kong. The original 23-item survey was based on an extensive literature review. It contains survey items from previous studies that examined nurses’ perceptions of their current practices and the essential components of effective shift-to-shift nursing handovers [22, 23]. The survey items have been psychometrically validated in the Hong Kong context [11, 13].

The NHPQ consists of a series of statements about nurses’ overall perceptions of handovers and their experiences of clinical handover in a bilingual context. Specifically, the statements focus on the nurses’ views of the presentation, organisation, comprehension and dissemination of patient information and their perceptions of ISBAR. The 23 items cover four features of nurses’ perceptions of clinical handovers:

- (a) the participants’ demographic background (7 items);
- (b) views on the quality of information exchanged in their current handover experiences (6 items);
- (c) perceptions of how information is presented during their own handover experiences in a bilingual context (13 items);
- (d) willingness to use the ISBAR protocol to perform handovers in a bilingual context (4 items)

Responses to the NHPQ items are on a 4-point Likert scale, where 1 indicates strong disagreement, 2 = disagree, 3 = agree and 4 indicates strong agreement. Of the 23 items, 8 (questions 3, 4, 8, 10, 17, 18, 21 and 22) are worded in a negative manner to reduce the likelihood of acquiescence bias. The scores for these items are reversed prior to statistical analysis. Due to the small sample size, the team performed descriptive statistical analysis. Means, standard deviations (S.D.) were evaluated based on the survey responses before and after the intervention.

## Results

### Participants’ demographic backgrounds

**Fig. 1:**
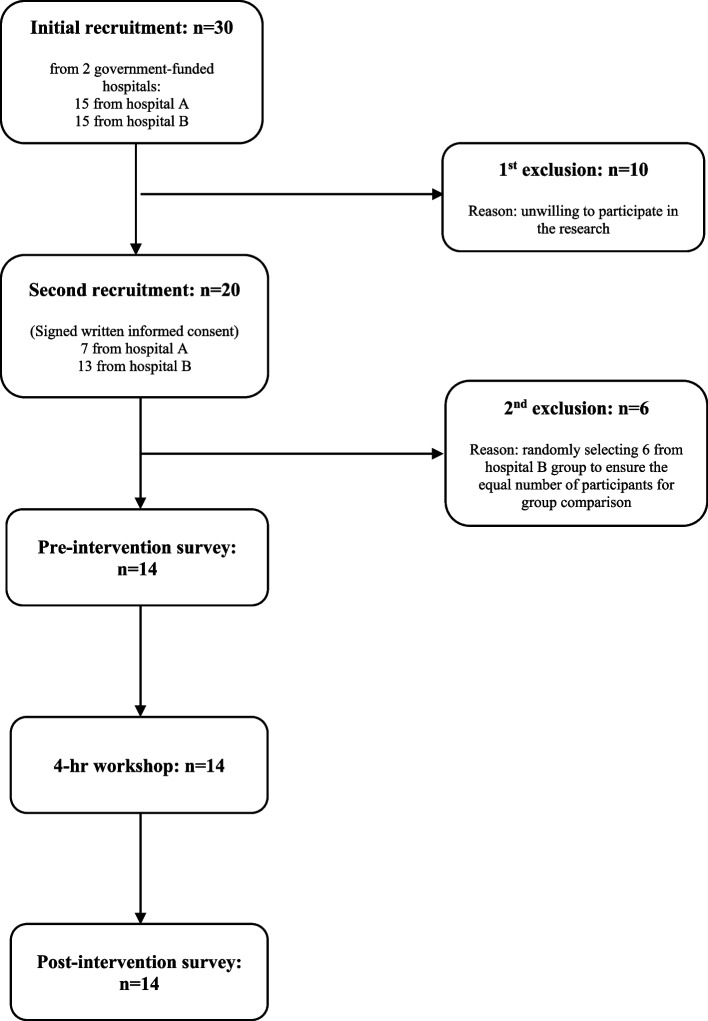
Flow chart of the recruitment process of nurses for the communication intervention

As shown in Fig. 1, 30 nurses altogether from the two hospitals were invited for the communication intervention. Nurses that respectfully decline the invitation are the two hospitals are eight and two respectively. To ensure an equal number of participants from both hospitals, six nurses from the second hospital were excluded. The remaining fourteen registered nurses gave written informed consent, completed the NHPQ, and their responses were submitted anonymously to the researchers via an online Google form. Eleven of the participants were female and three were male. Their ages ranged between 20 and 34. Ten had a Bachelor’s degree and four had a postgraduate diploma. Ten had worked for 2 to 5 years and the remaining staff worked at least 1 year. None had received professional training about handover communication skills before the simulation-based training workshop. Therefore, this study provided an opportunity for these nurses to improve their handover communication and to evaluate the proposed handover training protocol. Table 3 summarises the participants’ perceptions of their handover experiences before and after the intervention.

- (a) Participants’ views on the quality of the information exchanged during handovers.

**Table 3:** Participants’ perceptions of their own handover experiences before and after the intervention on 5-Likert scale (*n* = 14)

No	Item(s)	Mean (S.D.)	
		Pre-intervention	Post-intervention
Part A. Participants’ views on the quality of the information exchanged during handoverss			
1	I have been provided with adequate information about patients in my care	1.86 (.36)	3.23 (.44)
11	The information I received was up to date	2.93 (.73)	3.38 (.65)
13	I had the opportunity to ask questions about things I did not understand during a handover process	2.93 (.47)	3.31 (.63)
14	I was asked to clarify if I had any questions about the information received	3.14 (.53)	3.38 (.51)
16	I received adequate information about nursing care during the handover, for example, mobility, nutrition/hydration, pain	2.86 (.53)	3.23 (.60)
18	From my observations, important information about medication is often not given during the handover, for example, withheld medication, allergy, availability	2.64 (.74)	2.38 (.65)
Part B. Participants’ perceptions of an effective handover in a bilingual context			
2	The handover information was presented in a systematic and organised way	2.93 (.73)	3.46 (.52)
5	Charts were available during the handover to clarify the information provided to me	2.71 (.61)	3.38 (.65)
7	The way in which information was provided to me was easy to follow	2.79 (.43)	3.08 (.49)
9	I think effective communication skills should be used during a handover, such as clear speech that is not too fast	3.43 (.65)	3.69 (.48)
6	I used charts to review patient care during the handover, such as drug charts, vital signs, patient allergy information, FBC results	2.86 (.49)	3.38 (.65)
15	As a result of the handover, I have a clear understanding of the plan (diagnosis, treatment, discharge) for the patient(s)	3 (.39)	3.23 (.60)
Part C. Participants’ willingness to use the ISBAR protocol to perform handover in a bilingual context			
19	I believe that using ISBAR will help me to improve communication skills with my co-workers	2.93 (.47)	3.69 (.48)
20	I believe that using ISBAR will increase the quality of patient care and safety	3.14 (.53)	3.69 (.48)
21	I think that ISBAR is time-consuming	2.07 (.47)	2.31 (.85)
22	I think that ISBAR is not easy to implement in my handovers	2.29 (.47)	2.08 (.76)

Part A of Table 3 summarises the survey responses related to the participants’ perceptions of their own handover experiences. For Item 1, about receiving adequate information about patients during handover, 14.3% of the respondents were not satisfied with their practice before the training. Despite the small number of nurses recruited, the training nonetheless showed effective results. There was a noticeable increase in the nurses’ scores for this item, with all of them expressing general or strong agreement, and a 23.1% increase in the number of participants expressing strong agreement. This result suggests that the training provided these nurses with essential skills to increase their perception in providing adequate information about patients to their colleagues at handover.

Before the training, 28.6% of the respondents expressed that they did not receive up-to-date information at handover (Item 11). After the training, there was a 20.9% decrease in disagreement and a 24.8% increase in strong agreement with the statement. This result suggests that the participants realised the importance of presenting and receiving up-to-date information about patients, leading to effective handover practices. Clarification in handover practice is essential for nurses to obtain accurate information to avoid misunderstanding, allowing important patient information to be exchanged among nursing teams and thus enhancing collaboration between colleagues. Our results revealed a 17.1% increase in the number of participants expressing strong agreement that they had opportunities for clarification after the training (Item 14). At the pre-training stage, over 50% of the respondents indicated that they did not receive important information about medication during handovers (Item 18). After training, there was an 11% decrease after training in this perception, suggesting that after the training the participants realised the importance of providing information about medication during handovers: nursing staff can get the details they need when information is presented using relevant guidelines or protocols.

In summary, it is possible to observe two important differences between the participants’ responses before and after the training. First, their perceptions of providing and receiving adequate information about the patients, nursing care and medication during handovers had changed after the training. The survey responses to Items 1, 11, 16 and 18 indicate that after the training, these nurses realised the importance of providing and receiving up-to-date information. Second, the respondents’ perceptions of their opportunities to ask questions and gain clarification also changed. Any misunderstanding and lack of knowledge about a patient’s situation may lead to failure of nursing care. The responses to Items 13 and 14 suggest that after the training, the participants realised the importance of gaining mutual understanding with colleagues to obtain accurate information during handovers.

- (b) Participants’ perceptions of an effective handover in a bilingual context.

Part B of Table 3 summarises the survey responses indicating the participants’ perceptions of their own handover experiences. Before the training, 28.6% of the respondents indicated that they disagreed that information was presented in a systematic and organised way during the handover (Item 2). After the training, none of them indicated disagreement with the statement, and there was a large increase (from 21.4% to 46.2%) in the proportion of respondents who strongly agreed that handover information had been presented in a systematic and organised way. This result suggests that after the training, these nurses understood the importance of presenting information in a structured and organised way for a handover to be effective.

Before the training, the participants showed a lack of reliance on charts during handover (Item 5): 35.7% of them said that charts were not available to clarify patient information. After the training, there was a 39.1% increase in the number of participants who strongly agreed that using charts to clarify patient information is one way to achieve effective handover. When the participants were asked whether they had used charts to review patient care at handover (Item 6), a noticeable shift from before to after the training was observed. After the training, 92.4% of the participants reported that they decided to use charts to review patient care at handover, and there was a 31.9% increase in the number of respondents expressing strong agreement with the statement. This result suggests that the training encouraged these nurses to make good use of patient care charts to revisit and clarify patient information at handover. Moreover, most of the respondents (85.7%) agreed that they had a clear understanding of the plan for the patients before the training (Item 15). After the training, there was a 23.7% increase in the proportion of participants indicating strong agreement with the statement, suggesting that the participants had more confidence in obtaining a clear understanding of the plan, leading to effective handover practice.

In summary, the above results echo those described in the previous section on the change in the nurses’ perceptions of how information is presented during a handover, especially in terms of their strong agreement with each of the statements after the training. All of the respondents agreed with the application of effective communication skills such as speaking clearly and not too rapidly at handover. After the training, the nurses were more confident in using charts to review and present information in a systematic and organised way. Furthermore, the communication skills and ISBAR protocol introduced in the workshop seemed to help them to obtain a clear understanding of the nursing plan during the handover, leading to an effective handover practice.

- (c) Participants’ willingness to use the ISBAR protocol to perform handover in a bilingual context.

Part C of Table 3 summarises the survey responses relating to the participants’ willingness to use ISBAR when performing a handover. On Item 19, nearly 70% of the participants strongly agreed that the ISBAR protocol helped them to improve handover communication with their colleagues after the training, a 62% increase compared with the statistic before training. After the training, none of them disagreed with using ISBAR to promote better team-based communication at handover. These results suggest that the ISBAR protocol provides a good framework for promoting effective communication among nursing colleagues at handover. Furthermore, after the training, all participants agreed that using the ISBAR protocol could increase the quality of patient care and safety (Item 20). Nearly 70% of the respondents indicated strong agreement with the statement, a 47.8% increase from before to after the training. The participants’ responses suggested that their confidence in applying the ISBAR framework when providing clinical information at handover had increased, leading to better patient safety and care in a bilingual environment.

In summary, the questionnaire responses suggest that after the training, all of the respondents believed that the protocol could effectively increase the quality of patient care and safety and help them to improve their communication with their co-workers. Most of the participants agreed that the ISBAR protocol could be easily applied in their own handovers. All of the evidence indicates that after the workshop, the nurses had gained knowledge about the ISBAR framework and were willing to apply it in their own practice.

## Discussion

In this study, we developed, and implemented simulation-based training for effective nursing handover in a bilingual context. Although the number of participants was small, meaning that our findings may not be generalisable, the results indicate a dramatic change in the participants’ views from before to after training in the following areas: 1) the quality of information exchanged in their own handover experiences; 2) their perceptions of how information is presented during handovers in a bilingual context; and 3) their willingness to use an ISBAR protocol.

When evaluating the participants’ own handover experiences, a noticeable change could be seen in the level of satisfaction with the provision of adequate information after the training. For example, the nurses reported that they were given more opportunities to ask questions about and clarify patient information that they did not fully understand at handover. This process allows for readback between incoming and outgoing nurses to reach a mutual understanding of a patient’s current condition. This finding echoes the findings of previous research that the opportunity to ask questions is significantly correlated with improved handover quality, which the loci of research focusing on both bilingual communities within and outside Hong Kong [4, 7, 11–15]. The level of widespread research on the topic elicits further the importance of handover communication training for nursing staff and practitioners operating in clinical handovers, and that it should be implemented at a more universal level; observations from other research which were not covered in the training but nevertheless important and relevant report that a paucity of handover communication can result in handover quality inconsistency [5, 24], role confusion [25], and overall quality of patient care [26–28].

Given our research findings and the cited references, this suggests that the training we have implemented using simulation-based approach can provide nurses with essential skills to build up their confidence in providing adequate information about patients at handover. It appears that nurses do actively seek important information when conducting handovers [28], which further infers the utility of the training. Not only does it help them know what information they should look for, but also how to relay and communicate said information with others in their team—hence improving the quality of information exchanged and reducing occurrences for miscommunication.

In reference to the second point signposted, after the training, the participants’ perceptions of how information was presented during handover in a bilingual context indicated a change towards a more positive perception of their handover experiences. For example, they gained knowledge about and familiarity with using a structured flow to present information such as through the use of charts. This corresponds to how all of the participants believed that presenting information in a systematic and organised way would result in an effective handover. A previous study by Pun supports the participants’ positions, as the quality of handover practices depends on the degree of nurses’ understanding of the patient care plan [13]. Studies in the same context reflects on how such a training also aids staff to be more aware of the importance of interactions between relevant parties, and to be more actively engaged with their team members and each patient under their care [7, 11]. Therefore, we believe that increasing nurses’ confidence in obtaining a better and clearer nursing plan will enhance the quality of their own handover experiences.

When asked about their willingness to use an ISBAR protocol, the participants demonstrated a positive change and increased knowledge of the framework. For example, they expressed that the ISBAR protocol could help them to improve communication with their colleagues, leading to better patient safety and care in their bilingual clinical environment. This echoes that of previous research that explored the use of a handover protocol by nurses in a bilingual context [11, 13], finding that if nurses apply systematic handover protocols such as ISBAR, continuity of care is improved because the outgoing nurses can understand the explicit transfer of responsibility and incoming nurses can take more responsibility for engagement at handover. Similarly, in a monolingual clinical environment, the implementation of ISBAR protocol with attention to authentic interactive and informational skills was argued to be helpful for handover communication by supporting outgoing nurses to structure their information [4]. Other observed benefits of adopting the protocol include, a reduced miscommunication frequency with other staff [14], junior staff having improved confidence in performing handovers with their seniors [29], and improved nursing culture (for staff to be productive, respected, and have an increased sense of professionalism) [30].

In addition to the ISBAR protocol that views an effective handover process from five informational dimensions, this study also used the CARE-term protocol [8], which discloses handover from four staged speech acts during handover: connect, ask, respond, and empathise. The findings showed a positive change in realizing the importance of providing and receiving up-to-date information and obtaining mutual understanding with colleagues after the intervention. These changes can be related to the use of CARE-team protocol in that the mutual understanding construction between outgoing and incoming nurses through providing and restating timely and succinct summaries. This finding resonates with previous research that CARE protocol training in bilingual hospitals revealed significant outcomes in enhancing nurses’ ability to engage in clear, interactive, and informative handovers [9]. Moreover, the results demonstrated an improvement in the perceived importance of asking questions and gaining clarification during the handover process. This can be explained by the collaboration of two protocols that might help trainees reach a more comprehensive understanding of high-quality handover communication. For instance, the ISBAR stages help to specify when and what to ask at the “Ask” stage in the CARE-team protocol and the “Empathise” stage in the CARE-team protocol reminds outgoing and incoming nurses of how to ask. Hence, it can be safe to suggest an integration of these two protocols to further enhance the interactive frequency, effectiveness, and accuracy of handover in a bilingual context, which then promotes healthcare quality.

Using a simulation approach is advantageous to nursing handover practice. First, it serves as both a teaching strategy and an evaluation tool. Such an approach to professional development of nursing handover can improve patient safety, prepare new nurses and create innovative teaching strategies. Second, according to Nelson, using a simulation approach can give frontline and future nursing staff an opportunity to ‘practise on plastic’, which allows them to sharpen their skills and practise techniques such as using handover protocols [31]. Trainee nurses can practise in a non-threatening environment, which helps them to feel more prepared and more confident for the clinical portion of their course. Third, using a simulation approach can also address the theory–practice gap and transfer essential nursing skills and knowledge from the classroom to a real clinical setting [32]. It provides a situated learning approach that leads to increased confidence and transfer of learning to the real-world environment. Our findings echo those of a study by Lee and Lim, which found that self-efficacy and handover knowledge among nursing staff increased after a simulation-based training [19]. They also found that the participants’ handover performance improved after the training, confirming that using a simulation-based approach can enhance nurses’ handover experiences after training.

### Limitations

The participants’ perceptions had noticeably changed from before to after the training, and they had gained knowledge and willingness to use their communication skills during handovers—albeit that these observations come from a small participant sample size. It is for this reason that further investigation is needed to explore the persistence of the effects of the simulation-based training. Only fourteen participants were recruited for this study, and were from only two hospitals in Hong Kong. While a lot of research as cited in the discussion support the importance and effectiveness of our findings, they come from contexts outside of Hong Kong; further research in this area is warranted. Due to the small participant sample as mentioned, generalisation of the research findings should be made cautiously.

## Conclusion

Research on handover communication skills training in a bilingual context using a simulation approach is limited [14]. This study reports the participating nurses perceived their handover communication to have improved using simulation-based training. Using a simulation-based approach, the training build participants’ confidence and enhance their communication skills for better presenting and receiving patients’ information in an effective and organised way by using an ISBAR and CARE protocols. The nursing staff were more self-confident in their handover experiences, with a deeper understanding of using protocols after simulation-based training intervention.

We call for more research in this field, specifically related to the use of a simulation approach for training, and evaluation of clinical nursing handover practice with a specific focus on identifying avoidable communication issues that emerge from the bilingual clinical context, as well as issues that arise when using a standardised handover protocol such as ISBAR, to ensure safe and realisable handover communication among staff in a bilingual environment.

## Supplementary Information

**Additional file 1.** ISBAR protocol with examples of transition markers (adapted from Eggins et al. [8])

## Data Availability

All data generated or analysed during this study are included in this published article.

## Abbreviations

ISBAR: Introduction Situation Background Assessment Recommendation
NHPQ: Nurses Handover Perceptions Questionnaire
CARE: Connect Ask Respond Empathise
