# Supplementary material for: Using a simulation-based approach to promote structured and interactive nursing clinical handover: a pre- and post-evaluation pilot study in bilingual Hong Kong
Source: BMC Nurs. 2023 Feb 10;22:38. doi: 10.1186/s12912-023-01189-w (PMC9921344; doi:10.1186/s12912-023-01189-w)
Supplement: Supplementary file 1 — Additional file 1. ISBAR protocol with examples of transition markers (adapted from Eggins et al. [8]) [file 12912_2023_1189_MOESM1_ESM.docx]

Appendix

ISBAR protocol with examples of transition markers (adapted from Eggins et al. [8])

| **Transition** | **Marker** |
| --- | --- |
| When starting the **Identification** stage | Now, if you’re ready, moving on to the next patient ...  So, I’ve finished with patient X unless you have any questions. [wait a moment] OK, my next patient is patient Y. |
| When moving into the **Situation** stage | Now, the reason for patient Y’s admission on 12 December was ... |
| When moving into the **Background** stage | As for patient Y’s background, in 2012 he was diagnosed with ... |
| When moving into the **Assessment & Actions** stage | Now, to bring you up to date with how patient Y is today ...  Just to re-cap patient Y’s progress today ... |
| During the **Assessment & Actions** stage, to indicate substages | As far as mobility is concerned ... As for patient Y’s diet, he ... |
| During the **Recommendation**s stage | Now, as for patient Y’s care today, you will need to ... |
| During the **Readback** stage | Next, I’ll just summarise what you need to do for patient Y on your shift ... |
